# Toothache Drops

**Published:** 1880-12

**Authors:** 


### TOOTHACHE DROPS.

Powdered gum camphor, one ounce; chloral hydrate, one ounce. Rub them together in a wedgewood mortar until they liquify. Apply to the cavity on a small piece of cotton.—"*Hand Book of Private Formulas.*"

